# Analytic Design Technique for 2D FIR Circular Filter Banks and Their Efficient Implementation Using Polyphase Approach

**DOI:** 10.3390/s23249851

**Published:** 2023-12-15

**Authors:** Radu Matei, Doru Florin Chiper

**Affiliations:** 1 Faculty of Electronics, Telecommunications and Information Technology, “Gheorghe Asachi” Technical University of Iaşi, 700506 Iaşi, Romania; rmatei@etti.tuiasi.ro; 2 Institute of Computer Science, Iaşi Branch of the Romanian Academy, 700481 Iaşi, Romania; 3 Technical Sciences Academy of Romania (ASTR), 700050 Iaşi, Romania; 4 Academy of Romanian Scientists (AOSR), 030167 București, Romania

**Keywords:** 2D FIR filters, circular filters, analytical design, filter banks, polyphase decomposition, block filters

## Abstract

This paper proposes an analytical design procedure for 2D FIR circular filter banks and also a novel, computationally efficient implementation of the designed filter bank based on a polyphase structure and a block filtering approach. The component filters of the bank are designed in the frequency domain using a specific frequency transformation applied to a low-pass, band-pass and high-pass 1D prototype with a specified Gaussian shape and imposed specifications (peak frequency, bandwidth). The 1D prototype filter frequency response is derived in a closed form as a trigonometric polynomial with a specified order using Fourier series, and then it is factored. Since the design starts from a 1D prototype with a factored transfer function, the frequency response of the designed 2D filter bank components also results directly in a factored form. The designed filters have an accurate shape, with negligible distortions at a relatively low order. We present the design of two types of circular filter banks: uniform and non-uniform (dyadic). An example of image analysis with the uniform filter bank is also provided, showing that the original image can be accurately reconstructed from the sub-band images. The proposed implementation is presented for a simpler case, namely for a smaller size of the filter kernel and of the input image. Using the polyphase and block filtering approach, a convenient implementation at the system level is obtained for the designed 2D FIR filter, with a relatively low computational complexity.

## 1. Introduction

The technology and architecture of modern image sensors and sensing techniques have evolved dramatically in recent years, driven by the ever-demanding requirements and challenges of this field. For instance, aerial or satellite image sensors for remote sensing must provide clear and low-noise images, with high spatial resolution, either in visible, infrared or microwave domains. In order to provide accurate and relevant information, images acquired by sensors have to be pre-processed using various restoration and enhancement techniques. Various digital filters and filter banks may be used in image analysis and feature extraction tasks, for instance, to decompose the image into several subband components in order to extract relevant details, etc. These are also useful in the automotive field, for rapid feature extraction in real-time computer vision applications, for instance, in driver assistance systems and autonomous driving vehicles.

Along with the unprecedented development of the digital signal processing field, 2D filters have been thoroughly investigated by many researchers, owing to their essential applications in image processing, and various techniques for their design have been elaborated [1]. Analytical design methods rely on 1D prototypes with specified shapes and parameters; applying various frequency transformations, they lead directly to the desired 2D filters. The major advantage of the analytical approach is that a closed-form frequency response is derived, and the 2D filter results are parametric and therefore adjustable.

A large variety of 2D filters, both of FIR and IIR type, with various characteristics and shapes have been developed, with each type of filter having specific applications in the image processing field. One of the best-known methods, widely used in the design of 2D FIR filters with various shapes is the McClellan transform [2,3]. More recent papers approaching computationally efficient 2D FIR filter design techniques based on frequency transformations are [4,5]. The efficient, low-complexity design of 2D FIR filters and the implementation using Farrow structure are described in papers like [6,7]. Other relevant recent papers on efficient 2D filter design are [8,9,10].

Filters with circular-shaped frequency response have also been widely used owing to their capabilities in image analysis; various design techniques have been proposed for circular filters (CF) in early papers such as [11,12,13]. Circular filters find applications in texture segmentation and classification [14]. A recent advanced application of circular Gabor filters in SAR interferograms is described in [15].

Two-dimensional filter banks of various types were extensively used in important applications, like texture segmentation and classification or various feature extraction tasks. Such filter banks decompose the frequency spectrum of the image into a number of sub-bands. Two-dimensional filter banks are widely used in fundamental applications such as sub-band coding and compression of images and video sequences. Separable 2D filter banks are obtained by cascading 1D filter banks, and data are processed in each dimension separately. Compared to separable filters, filter banks with nonseparable 2D filters are more flexible and versatile, offering superior performance for imposed specifications. However, their design is substantially more difficult than for separable filter banks [16]. As detailed in the comprehensive review [16], the 2D filter banks currently used are mainly directional, with specific shapes in the frequency plane, such as square (diamond), parallelogram, wedge/fan filters, etc. Multidimensional stable, perfect reconstruction filter banks are also developed in [17].

Directional filter banks (DFBs) with an arbitrary number of sub-bands [18] or arbitrary frequency partitioning [19] have been proposed. A class of multiresolution DFBs is developed in [20]. Multidimensional DFB, multiscale pyramids and the surfacelet transform were introduced in [21]. A very recent application of DFBs was proposed in [22], namely fingerprint image quality assessment. The fingerprint image is decomposed into subbands using the DFB, and similarity between the different subbands is used to calculate the fingerprint image quality. Regarding methods to reduce computational complexity and increase processing speed, the fast block implementation of 2D digital FIR filters was proposed in early papers such as [23]. A high-performance 2D parallel block-filtering system for real-time applications was presented in [24]. The steerable pyramid, a well-known multiscale structure for image decomposition was proposed in the early paper [25]. More recent papers describe specific applications of other two important multiscale architectures, namely the Laplacian pyramid [26] and the wavelet pyramid [27].

Some very recent works propose advanced algorithms implemented on various convolutional neural networks to solve complex image-processing tasks. For instance, in [28], a novel deep-feature model has been proposed for coastal wetland classification using multisource satellite remote sensing data. In [29], multi-scale features from coarse-to-fine receptive field level are extracted, with applications in super-resolution. An advanced algorithm for effective pathology classification from hyperspectral medical images is proposed in [30]. A novel multi-focus image fusion method based on sparse representation and local energy is introduced in [31], which uses the shearlet transform to decompose the source images into low- and high-frequency sub-bands.

The first author of this paper has also proposed various analytical design techniques for 2D filters in previous works [32,33,34,35]. Directional IIR filters based on Gaussian and wide-band prototypes were designed in [32]. A useful application of the directional filters in [32] is the detection of straight lines with specified orientation from images; this feature extraction capability may be useful in the computer vision field. Adjustable, parametric 2D digital IIR filters with elliptical and circular symmetry are proposed in [33]. Two versions of circular IIR filter banks and their applications have been described in [34,35]. An efficient 2D FIR filter implementation based on a polyphase approach and block filtering is proposed in [36].

In this paper, an analytic design procedure is proposed for a particular class of 2D filter banks, namely 2D FIR Gaussian circular filter banks (CFBs). Two versions of CFBs will be designed, namely a uniform CFB and then a non-uniform (dyadic) CFB, each with a specified number of component filters. As a prototype, a 1D low-pass filter with a Gaussian frequency response and specified selectivity is chosen; its frequency response is easily approximated by a trigonometric polynomial, with an imposed precision, using a simple Fourier series expansion. By a simple shifting to a given peak frequency, the band-pass filters of the FB prototype are also derived. Once the prototype FB is obtained, a 1D to 2D frequency mapping derived from the McClellan transform is applied [2,3], which leads directly to the desired circular filters of the CFB. The non-uniform (dyadic) CFB is designed in a similar manner. The filters’ characteristics result in an accurate circular shape, with some distortions near the frequency plane margins. Next, as an application example, a grayscale test image is applied to the CFB, obtaining a set of subband images. Summing back all these images, the original input image is reconstructed almost perfectly, which suggests a potential use in an alternative subband coding scheme.

A novel, efficient implementation solution is also proposed for the 2D FIR filters of the designed CFB, which continues the method from previous work [36]. Our implementation uses a polyphase decomposition of a given 2D filtering operation with large kernel size and a block filtering with smaller size matrices.

The paper is organized as follows: Section 2 presents the proposed analytical design procedure, first deriving the uniform and non-uniform prototype FB, then applying the frequency mapping and obtaining the frequency responses of the 2D CFBs. In Section 3, an example of image analysis is given using CFB by decomposing it into subband images. The novel implementation technique based on the polyphase and block filtering approach is described in Section 4. Discussions regarding the computational complexity of the proposed implementation are included in Section 5. Finally, conclusions are drawn in the last section.

## 2. Analytical Design Technique for 2D Circular FIR Filter Banks

A novel analytical design procedure is proposed for a class of 2D FIR circular filters. This design technique starts from an imposed prototype with specified parameters (peak frequency, bandwidth), to which a 1D to 2D frequency transformation is applied, leading to the desired 2D filters. In order to obtain through frequency transformation, the desired 2D circular filter bank, first a 1D prototype filter bank must be derived. A Gaussian-shaped filter was chosen as prototype, due to its useful property of scalability on the frequency axis.

### 2.1. Approximation of the Gaussian FIR Filter Prototype Using Fourier Series

The Gaussian filter in the frequency domain has the well-known expression $G\left(\omega \right)=\exp\left(-\sigma^{2}\omega^{2}/2\right)$, where σ is the dispersion parameter; for a simpler form, easier to handle, the substitution $p=\sigma^{2}/2$, or equivalently $\sigma =\sqrt{2p}$, will be used. Thus the Gaussian low-pass filter function takes the more convenient form $G_{LP}\left(\omega \right)=\exp\left(-p\cdot \omega^{2}\right)$, where *p* will be referred to as selectivity or scaling parameter. Considering a periodic function with period 2π and regarding the LP Gaussian function as a generating pulse, the following expression $H_{LP}\left(\omega \right)$ will be easily obtained, which is the Fourier series expansion of the Gaussian $G_{LP}\left(\omega \right)$ up to a given order *N*:

(1) $$G_{LP}\left(\omega \right)=\exp\left(-p\cdot \omega^{2}\right)≅\frac{1}{2\sqrt{p\pi }}\cdot \left(1+2\cdot \sum_{n=1}^{N}\exp\left(-\frac{n^{2}}{4p}\right)\cdot \cos n\omega \right)=H_{LP}\left(\omega \right)$$

From this Gaussian LP prototype, a band-pass (BP) prototype is easily produced by shifting the Gaussian laterally around the frequencies $\pm \omega_{0}$:

(2) $$\begin{array}{c} H_{BP}\left(\omega \right)=H_{LP}\left(\omega -\omega_{0}\right)+H_{LP}\left(\omega +\omega_{0}\right)=\exp\left(-p\cdot {\left(\omega -\omega_{0}\right)}^{2}\right)+\exp\left(-p\cdot {\left(\omega +\omega_{0}\right)}^{2}\right)≅\\ \frac{1}{\sqrt{p\pi }}\cdot \left(1+2\cdot \sum_{n=1}^{N}\exp\left(-\frac{n^{2}}{4p}\right)\cdot \cos\;(n\omega_{0})\cdot \cos n\omega \right) \end{array}$$

Directly using Expressions (1) and (2) implemented in a Matlab routine, in the following section, the low-pass, band-pass and high-pass components of the desired FIR filter bank prototype are calculated.

### 2.2. Design of a Gaussian Uniform FIR Filter Bank Prototype

Next, a uniform filter bank prototype with 11 Gaussian components will be designed, namely one low-pass filter, nine band-pass filters and one high-pass filter. In this uniform FB, the peak frequencies are equally spaced on the frequency axis. A bandwidth is imposed for the nine band-pass components equal to B=π/10=0.1π, while the low-pass and high-pass filters will have each half of this bandwidth, namely B/2=π/20=0.05π. The *k*-th ideal Gaussian BP filter is produced by shifting the LP prototype to the frequency $\omega_{0,k}=k\cdot \omega_{0}$, and will have the following expression:

(3) $$G_{BP\;k}\left(\omega \right)=G_{LP}\left(\omega -k\omega_{0}\right)+G_{LP}\left(\omega +k\omega_{0}\right)=\exp\left(-p\cdot {\left(\omega -k\omega_{0}\right)}^{2}\right)+\exp\left(-p\cdot {\left(\omega +k\omega_{0}\right)}^{2}\right)$$

**Figure 1:**
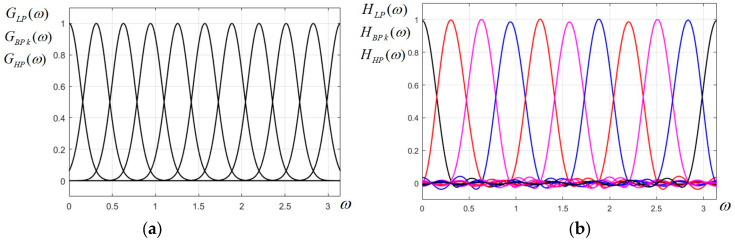
(**a**) Ideal Gaussian uniform FB prototype; (**b**) designed Gaussian uniform FB prototype for the 2D CFB.

At this point, the scaling parameter *p* for the imposed bandwidth needs to be calculated. In our case, the filter bandwidth is considered defined at 0.5 of the peak value (at 6 dB). Thus, the characteristics of any two adjacent filters will marginally overlap and will intersect at the value 0.5. Referring to the LP filter $G_{LP}\left(\omega \right)=\exp\left(-p\cdot \omega^{2}\right)$, the condition $G_{LP}\left(B/2\right)=\exp\left(-p\cdot B^{2}/4\right)=0.5$ is imposed, otherwise written $\exp\left(p\cdot B^{2}/4\right)=2$, from which the value for the scaling parameter *p* is obtained as $p=4\ln2/B^{2}$; since for our filter bank a bandwidth B=π/10 was imposed, the value $p=400\ln2/\pi^{2}≅28.1$ will be produced. The ideal uniform Gaussian filter bank is plotted in Figure 1a.

The filter selectivity is given by the scaling parameter value calculated before, namely *p* = 28.1, with the Fourier series truncated at a number of terms *N* = 15. The larger the number of terms taken into account, the smaller will be the distortions (ripple, etc.), but the filter matrices will be larger in size and will increase the implementation complexity.

Following the above design procedure, once specifying the desired number of filters of the FB and their peak frequencies, using Equations (1) and (2), the frequency responses of all the FB components are calculated. As an example, in our case of a uniform FB with 11 components, the frequency responses of a few filters of the 1D prototype filter bank are given below, in factored expression. First, the frequency response of an LP prototype expressed as a truncated Fourier series using (1) has the form:

(4) $$\begin{array}{l} H_{LP}\left(\omega \right)=0.055823+0.11066\cdot \cos\;\omega +0.107743\cdot \cos\;2\omega +0.103056\cdot \cos\;3\omega +0.096833\cdot \cos\;4\omega \\ +0.089382\cdot \cos\;5\omega +0.081049\cdot \cos\;6\omega +0.072197\cdot \cos\;7\omega +0.063177\cdot \cos\;8\omega +0.054309\cdot \cos\;9\omega \\ +0.045863\cdot \cos\;10\omega +0.038047\cdot \cos\;11\omega +0.031007\cdot \cos\;12\omega +0.024823\cdot \cos\;13\omega +0.019523\cdot \cos\;14\omega \\ +0.015083\cdot \cos\;15\omega \end{array}$$

which using trigonometric identities can be further expressed as:

(5) $$\begin{array}{c} H_{LP}\left(\omega \right)=247.118\cdot (\cos\omega +0.99492)(\cos\omega +0.95454)(\cos\omega +0.87546)(\cos\omega +0.76089)(\cos\omega +0.61554)\\ \cdot (\cos\omega +0.44535)(\cos\omega +0.25729)(\cos\omega +0.05906)(\cos\omega -0.14123)(\cos\omega -0.33537)\\ \cdot (\cos\omega -0.51542)(\cos\omega -0.67403)(\cos\omega -0.80475)(\cos\omega -0.90811)(\cos\omega -0.93698) \end{array}$$

As an example, the frequency responses of the first and last BP filter components of the bank are given below, the intermediate BP filters having similar forms:

(6) $$\begin{array}{c} H_{BP\;1}\left(\omega \right)=-98.8414\cdot (\cos\omega +0.99462)(\cos\omega +0.95194)(\cos\omega +0.86846)(\cos\omega +0.74782)(\cos\omega +0.59531)\\ \cdot (\cos\omega +0.41762)(\cos\omega +0.22253)(\cos\omega +0.01863)(\cos\omega -0.18508)(\cos\omega -0.37954)\\ \cdot (\cos\omega -0.55591)(\cos\omega -0.71234)(\cos\omega -0.77319)(\cos\omega -1.00157) \end{array}$$

(7) $$\begin{array}{c} H_{BP\;9}\left(\omega \right)=-98.8414\cdot (\cos\omega -0.99462)(\cos\omega -0.95194)(\cos\omega -0.86846)(\cos\omega -0.74782)(\cos\omega -0.59531)\\ \cdot (\cos\omega -0.41762)(\cos\omega -0.22253)(\cos\omega -0.01863)(\cos\omega +0.18508)(\cos\omega +0.37954)\\ \cdot (\cos\omega +0.55591)(\cos\omega +0.71234)(\cos\omega +0.77319)(\cos\omega +1.00157) \end{array}$$

Finally, the highest component of the FB is the high-pass (HP) filter, which formally has the peak frequency $\omega_{0}=\pi$:

(8) $$\begin{array}{c} H_{HP}\left(\omega \right)=-247.118\cdot (\cos\omega +0.93698)(\cos\omega +0.90811)(\cos\omega +0.80475)(\cos\omega +0.67403)(\cos\omega +0.51542)\\ \cdot (\cos\omega +0.33537)(\cos\omega +0.14123)(\cos\omega -0.05906)(\cos\omega -0.25729)(\cos\omega -0.44535)\\ \cdot (\cos\omega -0.61554)(\cos\omega -0.76089)(\cos\omega -0.87546)(\cos\omega -0.95455)(\cos\omega -0.99491) \end{array}$$

It can be observed that the component filters of the prototype FB whose central frequencies are symmetric with respect to the middle value ω=π/2 have symmetric zeros, as is well-known from filter theory. Therefore, the zeros of the HP filter are the zeros of the LP filter with a changed sign; the zeros of the 9th BP filter are the zeros of the first BP filter with a changed sign, etc. Since there is an odd number of filters, the middle filter, namely the 5-th BP filter, with central frequency $\omega_{0}=\pi /2$, has no pair, and its transfer function, as expected, has pairs of complementary zeros:

(9) $$\begin{array}{c} H_{BP\;5}\left(\omega \right)=-319.858\cdot (\cos\omega -0.99471)(\cos\omega +0.99471)(\cos\omega -0.95281)(\cos\omega +0.95281)(\cos\omega -0.87107)\\ \cdot (\cos\omega +0.87107)(\cos\omega -0.75362)(\cos\omega +0.75362)(\cos\omega -0.60689)(\cos\omega +0.60689)\\ \cdot (\cos\omega -0.43317)(\cos\omega +0.43317)(\cos\omega -0.34221)(\cos\omega +0.34221) \end{array}$$

Generally, the *k*-th band-pass component of the 1D filter bank can be expressed as the following product of first-order factors (where *N* is the filter order):

(10) $$H_{BP\;k}\left(\omega \right)=\xi_{k}\cdot \prod_{j=1}^{N}\left(\cos\omega {+a}_{j}\right)$$

The uniform Gaussian filter bank designed above is plotted in Figure 1b and it looks very similar to its ideal counterpart in (a), with a low level of ripple.

### 2.3. Design of a Gaussian Non-Uniform FIR Filter Bank Prototype

In image analysis, mainly in multirate signal processing, non-uniform filter banks are also currently used. Next, using the method described in Section 2.1 a non-uniform, more specifically a so-called dyadic filter bank will be designed. Such an FB has the property that the bandwidths of the component filters increase proportionally to their peak frequencies, such that generally the ratio between bandwidth and peak frequency remains constant; these filters are also known as constant-Q filter banks.

For our design example, it is considered that the filter bandwidths increase by a factor of 2 from low to high frequencies. An FB with five filters will be designed here: one LP filter, three BP filters, and one HP filter. Specifying the peak frequency of the 3rd BP filter as $\omega_{03}=\pi /2$, the following peak frequencies $\omega_{0\;k}$ and bandwidths $B_{0\;k}$ are easily found for the five filters, respectively: $\omega_{0\;0}=0$, $B_{0\;0}=\pi /22$ (LP); $\omega_{0\;1}=\pi /11$, $B_{0\;1}=\pi /11$ (BP1); $\omega_{0\;2}=5\pi /22$, $B_{0\;2}=2\pi /11$ (BP2); $\omega_{0\;3}=\pi /2$, $B_{0\;3}=4\pi /11$ (BP3); $\omega_{0\;4}=\pi$, $B_{0\;4}=7\pi /22$ (HP). Using the same Formulas (1) and (2) as before, the frequency responses of the component filters are easily found as factored trigonometric polynomials. Unlike the previous case of uniform FB, for this nonuniform FB the higher filters have increasing bandwidths; being less selective, they can be approximated with polynomials of lesser order, therefore their implementation complexity will be significantly lower. The same marginal overlapping between filters at exactly 0.5 was considered. For instance, for the most selective filter (LPF) the parameter *p* results as $p=\ln2/{\left(\pi /22\right)}^{2}≅34$; this filter can still be approximated by truncating the Fourier series at order *N* = 15, as before; the ripple (“ringing”) in the stopband will be a little higher, but still acceptable. The following approximations for the frequency responses of the five Gaussian filters were derived:

(11) $$\begin{array}{c} H_{LP}\left(\omega \right)=322.53\cdot (\cos\omega +0.99491)(\cos\omega +0.95448)(\cos\omega +0.87527)(\cos\omega +0.76053)(\cos\omega +0.61495)\\ \cdot (\cos\omega +0.4445)(\cos\omega +0.25615)(\cos\omega +0.05762)(\cos\omega -0.14297)(\cos\omega -0.3374)\\ \cdot (\cos\omega -0.51771)(\cos\omega -0.67653)(\cos\omega -0.80734)(\cos\omega -0.90613)(\cos\omega -0.95148) \end{array}$$

(12) $$\begin{array}{c} H_{BP1}\left(\omega \right)=-261.27\cdot (\cos\omega +0.99507)(\cos\omega +0.95596)(\cos\omega +0.87933)(\cos\omega +0.76831)(\cos\omega +0.62742)\\ \cdot (\cos\omega +0.46239)(\cos\omega +0.2799)(\cos\omega +0.08752)(\cos\omega -0.10714)(\cos\omega -0.29616)\\ \cdot (\cos\omega -0.47196)(\cos\omega -0.62751)(\cos\omega -0.76157)(\cos\omega -0.81472)(\cos\omega -1.00137) \end{array}$$

(13) $$\begin{array}{c} H_{BP2}\left(\omega \right)=9.186\cdot (\cos\omega +0.98714)(\cos\omega +0.88634)(\cos\omega +0.69593)(\cos\omega +0.43711)\\ \cdot (\cos\omega +0.14021)(\cos\omega -1.00605)(\cos\omega -1.05338)((\cos\omega {)}^{2}-0.38658\cdot \cos\omega +0.05005) \end{array}$$

(14) $$H_{BP3}\left(\omega \right)=0.9531\cdot ((\cos\omega {)}^{2}+2.018676\cdot \cos\omega +1.024286)((\cos\omega {)}^{2}-2.018676\cdot \cos\omega +1.024286)$$

(15) $$H_{H\;P}\left(\omega \right)=-0.2117\cdot (\cos\omega -1.00109)((\cos\omega {)}^{2}-2.02723\cdot \cos\omega +1.68236)$$

**Figure 2:**
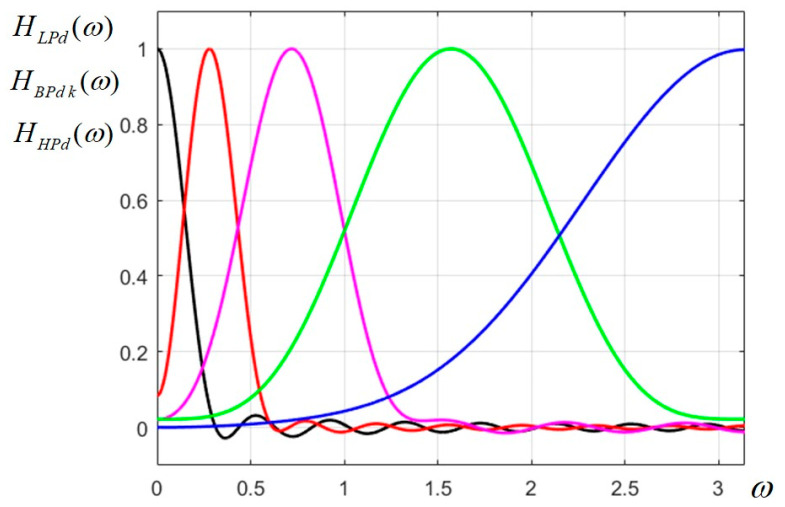
Designed Gaussian nonuniform (dyadic) filter bank prototype for the 2D CFB.

The characteristics of this non-uniform FB are plotted in Figure 2. 

As mentioned, only the most selective filters (LP and BP1) have visible ripple, while the others have no ripple at all.

As a further remark, in previous papers [32,33,34,35], various 2D filters were designed using another efficient procedure, namely the Chebyshev series, which has the advantage of yielding a uniform and efficient approximation for a given function, with equal error along the whole specified range of values. The symbolic calculations are performed in the MAPLE software (version MAPLE 2018), and a change of frequency variable is first required, before effectively deriving the approximation. However, the major drawback of this method is that it is not parametric; it does not have a closed form as in the case of the Fourier series method, therefore is more laborious; for each specified value of selectivity parameter *p*, the calculation must be carried out in a symbolic calculation software. Therefore, in this paper, the Fourier series approximation was preferred.

### 2.4. Gaussian Circular FIR Filter Bank Obtained Using Frequency Transformation

Once specified a convenient 1D prototype with the frequency response $H_{p}(\omega )$, a 2D circular filter $H(\omega_{1},\omega_{2})$ is produced by applying to the given prototype the 1D to 2D frequency transformation $\omega \to \sqrt{\omega_{1}^{2}+\omega_{2}^{2}}$:

(16) $$H(\omega_{1},\omega_{2})=H_{p}\left(\sqrt{\omega_{1}^{2}+\omega_{2}^{2}}\right)$$

The function $\cos\sqrt{\omega_{1}^{2}+\omega_{2}^{2}}$ is described by the 3×3 centrally symmetric matrix:

(17) $$C=\left[\begin{array}{ccc} 0.125 & 0.25 & 0.125\\ 0.25 & -0.5 & 0.25\\ 0.125 & 0.25 & 0.125 \end{array}\right]$$

and can be approximated by the following expression, which is a simple particular case of the McClellan transform, currently used in 2D FIR filter design [2,3,36]:

(18) $$\cos\sqrt{\omega_{1}^{2}+\omega_{2}^{2}}≅C(\omega_{1},\omega_{2})=-0.5+0.5(\cos\omega_{1}+\cos\omega_{2})+0.5\cos\omega_{1}\cos\omega_{2}$$

The Expression (18) is in fact the discrete space Fourier transform (DSFT) of the matrix **C**. Next, a zero-phase FIR filter $H_{P}(\omega )$ is considered, whose frequency response is given by the trigonometric polynomial expression [36]:

(19) $$H_{P}(\omega )=b_{0}+2\sum_{k=1}^{R}b_{k}\cos k\omega$$

At this point, the trigonometric identities for coskω (k=1…R) can be used, and thus the following polynomial expression is produced in powers of cosω [36]:

(20) $$H_{P}(\omega )=c_{0}+\sum_{k=1}^{R}c_{k}(\cos\omega {)}^{k}$$

where (19), (20) $b_{0},b_{k},c_{0},c_{k}$ are polynomial coefficients. Applying frequency mapping (18), the frequency response of the 2D circular filter will become [36]:

(21) $$H(\omega_{1},\omega_{2})=H_{P}\left(\sqrt{\omega_{1}^{2}+\omega_{2}^{2}}\right)=c_{0}+\sum_{k=1}^{R}c_{k}\cdot C^{k}(\omega_{1},\omega_{2})$$

where $C(\omega_{1},\omega_{2})=\cos\sqrt{\omega_{1}^{2}+\omega_{2}^{2}}$ as given in (18).

Therefore, by a straightforward substitution of cosω by the circular cosine function $C(\omega_{1},\omega_{2})=\cos\sqrt{\omega_{1}^{2}+\omega_{2}^{2}}$ in the prototype $H_{P}(\omega )$, the 2D filter frequency response is produced directly. Next, supposing that the frequency response $H_{P}(\omega )$ is decomposed into first-order and second-order factors in variable cos ω, and achieving the above substitution in all factors of $H_{P}(\omega )$, the circular filter frequency response $H(\omega_{1},\omega_{2})$ is finally derived in factored form:

(22) $$H(\omega_{1},\omega_{2})=k\cdot \prod_{i=1}^{n}\left(C+b_{i}\right)\cdot \prod_{j=1}^{m}\left(C^{2}+b_{1j}\cdot C+b_{2j}\right)$$

where *C* is a concise notation for the two-variable function $C(\omega_{1},\omega_{2})$ and *k* is the constant resulting from factorization. Since the specified prototype is expressed as a product of elementary factors, the circular filter frequency response will also become directly factored, which is an essential advantage in actual implementation. Thus, the large kernel **H** corresponding to $H(\omega_{1},\omega_{2})$ can be expressed simply as a discrete convolution of small matrices (of size 3×3 or 5×5):

(23) $$H=k\cdot (C_{1}*\ldots *C_{i}*\ldots *C_{n})*(D_{1}*\ldots *D_{j}*\ldots *D_{m})$$

The matrix expression (23) is related to the factored frequency response (22). Using the 3×3 matrix **C** in (17) and considering also (22), each of the matrices $C_{i}$ of size 3×3 in (23) is derived by adding coefficient $b_{i}$, which appears in the first-order factors in (22), to the center element in matrix **C**. Thus, the matrix $D_{j}$ (5×5) becomes:

(24) $$D_{j}=C*C+b_{1j}\cdot C_{1}+b_{2j}\cdot C_{0}$$

where $C_{0}$ is a null matrix of size 5×5 with central element of value one; $C_{1}(5\times 5)$ is produced by the boarding matrix **C** (size 3×3) with zeros; here the symbol * denotes convolution.

Thus, the frequency response of each CFB component is directly derived by substitution. Correspondingly, the overall kernel matrix H of the filter will be given by an expression similar to (23), but in our particular case with only first-order factors, as in (10), it becomes:

(25) $$H=\xi_{k}\cdot (C_{1}*\ldots *C_{i}*\ldots *C_{n})$$

The filters of the designed 1D prototype filter bank are of order 15; it follows that the corresponding 2D circular filters derived through the above transformation have kernel matrices relatively large, of size 31 × 31. Such a large matrix will be implemented efficiently using a polyphase approach described in Section 4.

As a remark, all the component filters of the designed FB are non-separable, except the LP filter. Indeed, it is easy to see that the circular LP Gaussian filter is separable as a product of two Gaussian LP filters on the two frequency axes:

(26) $$\exp\;\left(-p\cdot (\omega_{1}^{2}+\omega_{2}^{2})\right)=\exp\;\left(-p\cdot \omega_{1}^{2}\right)\cdot \exp\;\left(-p\cdot \omega_{2}^{2}\right)$$

A very important advantage of the proposed FB is that the filters’ transfer functions are real-valued (zero-phase), therefore they will not introduce any phase distortions; this will be visible in the simulation results given in the following section.

**Figure 3:**
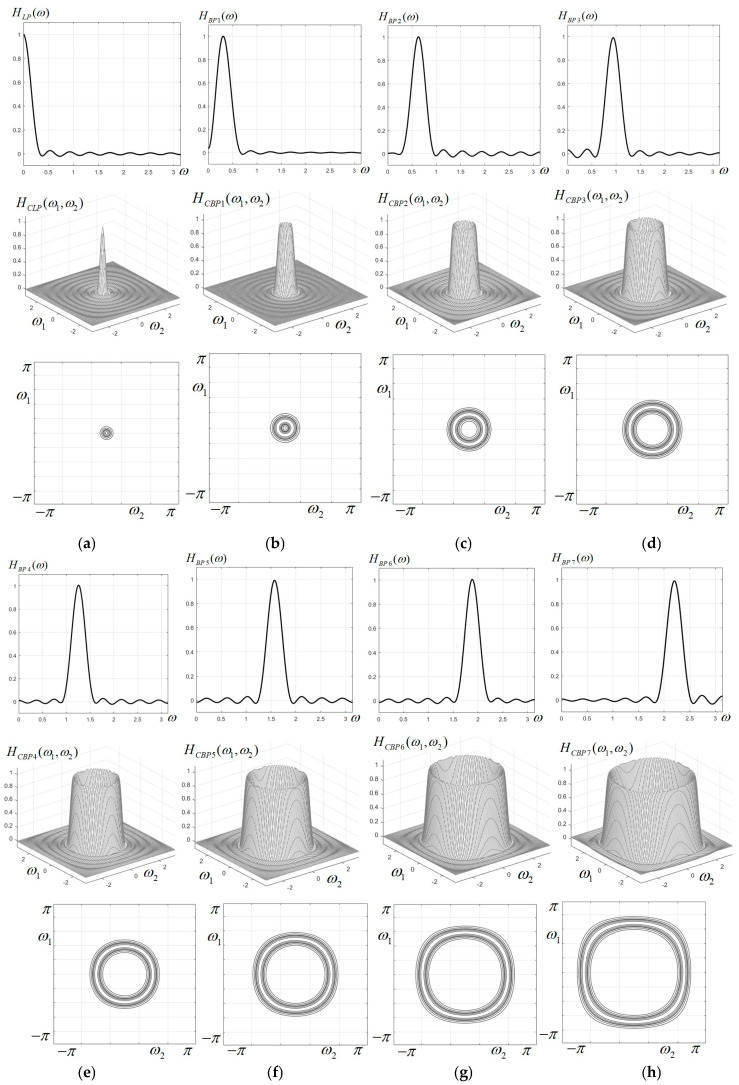
1D prototypes, characteristics and contour plots for the first eight components of the circular filter bank; (**a**) low-pass filter; (**b**–**h**) band-pass filters BP1–BP7.

**Figure 4:**
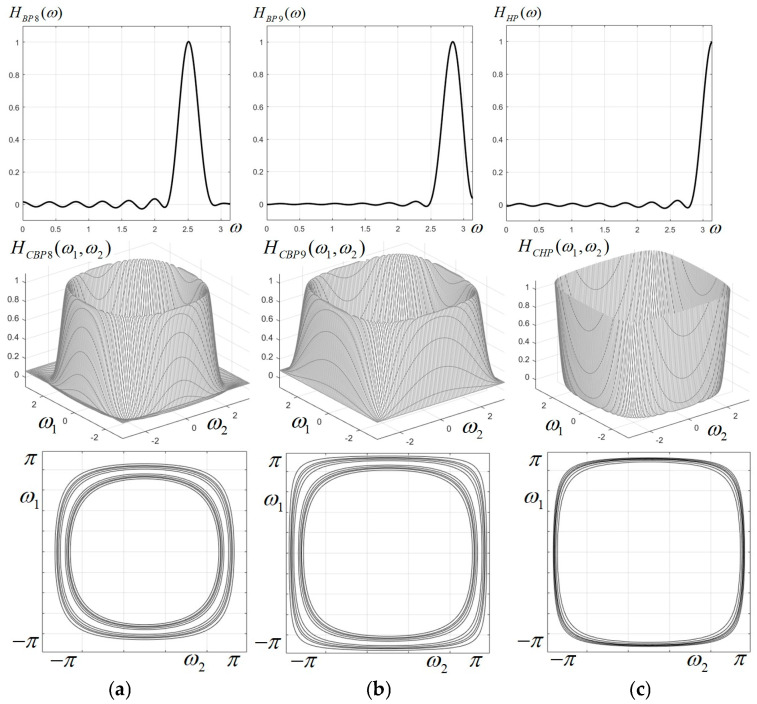
1D prototypes, characteristics and contour plots for the last three components of the circular filter bank; (**a**,**b**) band-pass filters BP8, BP9; (**c**) high-pass filter.

The 1D prototypes, frequency characteristics and corresponding contour plots for all 11 filters of the circular filter bank are displayed in Figure 3 and Figure 4. It is easily observed that up to the 6th band-pass filter, the characteristics are visually almost perfectly circular. For the higher band-pass filters, the characteristics have a more pronounced deviation from circularity, tending to the shape of a rounded square. The filter with the highest frequency ($\omega_{0}=\pi$) has almost a square shape. This effect of distortion from circularity is well known when applying the frequency mapping (18), the simplest form of the McClellan transform, and could be corrected only by using a more accurate approximation of the circular cosine; however, this would imply a higher complexity of the filters (larger kernel matrices) and a more difficult implementation.

**Figure 5:**
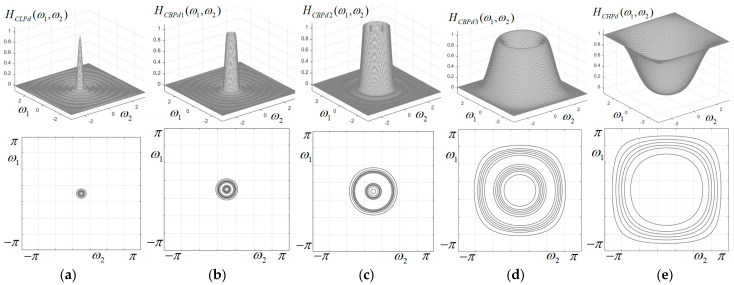
Characteristics and contour plots of the components of the dyadic CFB; (**a**) LP filter; (**b**–**d**) BP filters; (**e**) LP filter.

The characteristics and contour plots of the five component filters of the non-uniform (dyadic) CFB derived from the 1D prototype filters designed in Section 2.3, with frequency responses given by (11)–(15) are displayed in Figure 5, and it can be observed that they have a good circular symmetry.

## 3. Image Analysis Using the Designed Circular Filter Banks

**Figure 6:**
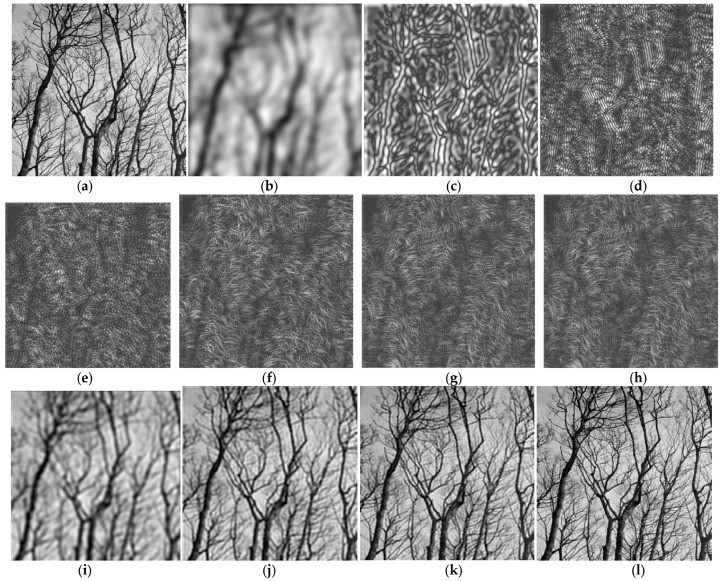
Image analysis using the uniform circular FB: (**a**) original “Trees” image; (**b**) LP filtered; (**c**–**g**) BP filtered with BPF1, BPF2, BPF3, BPF4, BPF5, respectively; (**h**) HP filtered; (**i**–**k**) recovered image by summing the first two, three and four components; (**l**) recovered image by summing all 11 components (sub-band images).

In this section, examples of image analysis using the uniform and dyadic CFBs designed before are presented. First, the grayscale test image in Figure 6a is considered, of size 399×399 pixels, representing a group of trees without foliage; this image was chosen as it has a lot of fine details, represented by the tree ramifications into thinner and thinner twigs. This image is filtered by applying all the 11 components of the designed uniform CFB (one LP filter, nine BP filters, one HP filter); it can be considered that our test image is decomposed into sub-bands using the analysis CFB designed before.

The original image is displayed in Figure 6a. The image obtained at the output of the narrow LP filter is (b), and it can be observed that it is very blurred, the fine details (thin twigs) are no longer visible. The images obtained from the first five BP filters are shown in (c–g), respectively, and contain details corresponding to the selected bandwidth. The image (h) is produced at the output of the HP filter and contains the highest frequencies, corresponding to the finest details.

The original image was converted into “double” format and its pixel values were rescaled to the range [0, 1] for MATLAB processing. The image produced at the output of LPF has the overall mean pixel value 0.529; for all the other 10 images (produced at the outputs of BP filters and HP filter), the mean pixel value is very close to zero, as expected, since these filters eliminate the zero-frequency component corresponding to mean value. In Figure 6i–l, it is shown how the original image is reconstructed by adding the component images into which it was decomposed. Thus, image (i) is produced by adding the first two components (LP and BP1); image (j) is produced as a sum of the first three components (LP, BP1, BP2); image (k) is produced by adding the component BP3. Finally, by summing all the 11 components, the image (l) is produced, which visually is very similar to the original image, showing all the fine details very clearly.

These simulations prove that the designed CFBs (uniform and non-uniform) could be practically used as analysis filter banks for decomposing a given image into sub-band images. However, the rigorous mathematical conditions required will have to be further investigated in future work.

The energy of each component sub-band image can also be evaluated using the well-known formula $E_{k}=\sqrt{\sum_{i=1}^{M}\sum_{j=1}^{N}p_{ij}}$, where the image is of size *M × N* and $p_{ij}$ is the current pixel value; the expression of the relative energy can also be given as a percentage:

(27) $$E_{R\;k}=\frac{100}{M\cdot N}\cdot \sqrt{\sum_{i=1}^{M}\sum_{j=1}^{N}p_{ij}}\;\left(\%\right)$$

**Figure 7:**
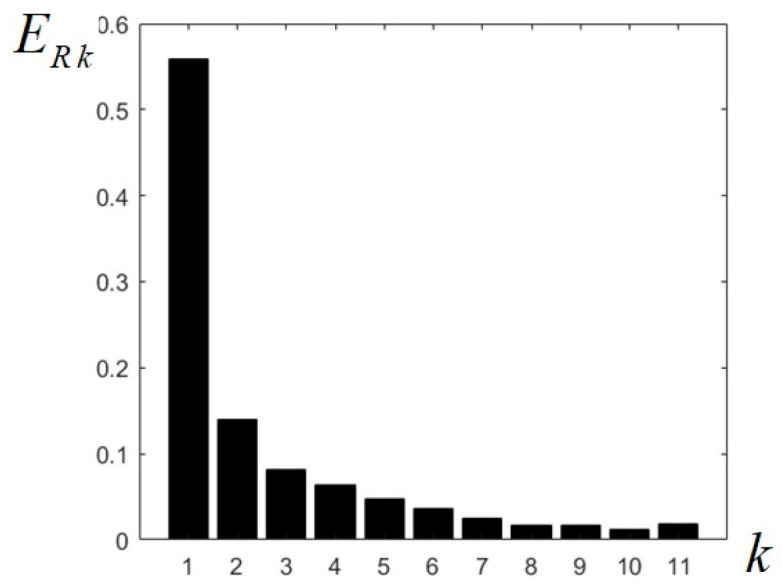
Relative energies calculated for the 11 images resulting at the output of the uniform circular filter bank.

Calculating the energies of the 11 filtered images resulting at the output of the designed CFB, the values given in Table 1 are easily found; summing these values, it can be verified that they add up to approximately 1 in normal values, or 100% in percentages. It can be observed that almost 56% of the image energy is contained in the low-pass component (in the frequency domain around zero, with radius 0.1π), while almost 85% is contained in the first four components (within a 0.4π radius), at the output of LP filter and first three BP filters. The relative energies of the sub-band images decrease almost uniformly; as an exception, ER9 > ER8, and ER11 > ER8, ER9, ER10. The highest frequencies in the image give less than 2% of the total image energy. These relative energy values are summarized in Table 1 and represented graphically in the chart from Figure 7.

As a remark, the designed circular filter banks are rotation invariant; the image spectrum is separated into concentric, ring-shaped regions, with frequencies increasing while image energy is generally decreasing, from the center to the margins of the frequency plane. Due to rotational invariance, the decomposition coefficient and energy in each subband remain more or less constant. This property is very useful in specific feature extraction and classification tasks in image processing.

**Figure 8:**
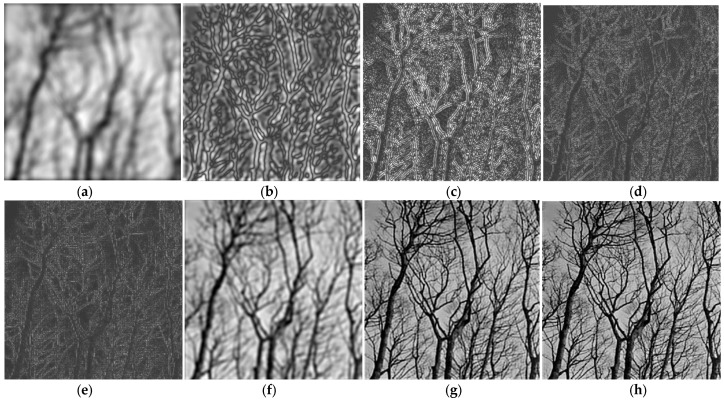
Image analysis using the dyadic circular FB: (**a**) LP filtered; (**b**–**d**) BP filtered with BPF1, BPF2, BPF3, respectively; (**e**) HP filtered; (**f**,**g**) recovered image by summing the first two and three components, respectively; (**h**) recovered image by summing all five component.

A similar experiment was performed using the dyadic circular filter bank with five components shown in Figure 5, applied on the same grayscale test image, for comparison. The filtered images obtained at the output of the LP filter, three BP filters and HP filter are displayed in Figure 8a–e. As in the previous example, the original image is then reconstructed by adding the first two and three sub-band images, then all the five sub-band images, as shown in Figure 8f–h. Summing up all the sub-band images leads to an image very similar to the original one.

**Figure 9:**
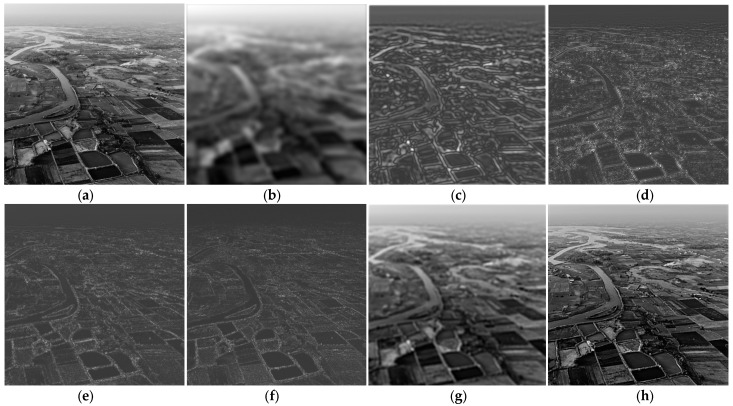
Image analysis using the dyadic circular FB: (**a**) “Fields” test image; (**b**) LP filtered; (**c**–**e**) BP filtered with BPF1, BPF2, BPF3, respectively; (**f**) HP filtered; (**g**) recovered image by summing the first two components; (**h**) recovered image by summing all five components.

An additional experiment was performed using the same dyadic CFB, applied on another grayscale test image (“Fields”), of size 699 × 699, showing an aerial view of a rural landscape with fields and a river (Figure 9a). The filtered images obtained at the output of the LP filter, three BP filters and HP filter, respectively, are displayed in Figure 9b–f. As in the previous examples, the original image is then reconstructed by adding the first two sub-band images (Figure 9g), then all the five sub-band images, as shown in Figure 9h. Summing up all the five sub-band images yields an image very similar to the original one. Table 2 displays the relative sub-band energies, calculated for both test images, namely “Trees” and “Fields”. Again, most of the image energy is contained in the lowest sub-band (corresponding to the LPF); however, the energy distribution clearly depends on the particular image, as was expected, an can be considered a numerical indicator characterizing the sub-band decomposition of a given image.

Regarding the noise suppression issue, the authors did not intend to investigate it in this paper. Of course, noise removal is a very important task in image enhancement and restoration. Considering the nature of the noise (Gaussian, salt-and-pepper, speckle noise, etc.), a specific type of filter should be chosen to remove it optimally. Anyway, noise removal should be achieved before any further image analysis. For the proposed CFB, since the image spectrum is partitioned into ring-shaped regions corresponding to sub-band images, if the original image was affected by some type of noise, it would be distributed more or less evenly in the sub-band images, mainly in higher frequency bands. Therefore, it would have to be eliminated separately from each sub-band component image, which may be a more difficult task. This issue remains to be studied in future work on this topic.

## 4. Polyphase Implementation of the Designed 2D Circular FIR Filters

In the following, a low-complexity implementation is proposed for the 2D FIR circular filter bank previously designed, relying on a polyphase structure of a 2D filtering task with a convolution kernel of relatively large size (31×31). In order to achieve convolution with such a large kernel, a block processing technique [24,25] and a polyphase decomposition approach will be employed.

As a first step, using sub-expression sharing techniques, a 2D filtering algorithm with a 4 × 4 kernel was elaborated, which is detailed as follows. The kernel of the filter resulting from the design and the input image are decimated by factors 3 and 5, respectively; the polyphase filtering approach is subsequently applied. Using this technique, three output component images are derived, namely $Y_{0}$, $Y_{1}$, $Y_{2}$, given by Equations (28)–(30):

(28) $$\begin{array}{l} Y_{0}=\left[\begin{array}{cccc} A_{0} & A_{0} & A_{0} & A_{0}\\ O_{4\times 10} & A_{0} & O_{4\times 10} & O_{4\times 10}\\ O_{4\times 10} & O_{4\times 10} & A_{0} & O_{4\times 10}\\ O_{4\times 10} & O_{4\times 10} & O_{4\times 10} & A_{0} \end{array}\right]\times diag\left(\left[\begin{array}{cccc} O_{10\times 4} & O_{10\times 4} & O_{10\times 4} & A_{1}\\ O_{10\times 4} & O_{10\times 4} & A_{1} & A_{1}\\ O_{10\times 4} & A_{1} & O_{10\times 4} & A_{1}\\ A_{1} & O_{10\times 4} & O_{10\times 4} & A_{1} \end{array}\right]H^{T}\right)\times \\ \;\times \left[\begin{array}{ccccccc} A_{2} & -A_{2} & -A_{2} & -A_{2} & O_{10\times 7} & O_{10\times 7} & O_{10\times 7}\\ O_{10\times 7} & A_{2} & O_{10\times 7} & O_{10\times 7} & O_{10\times 7} & O_{10\times 7} & O_{10\times 7}\\ O_{10\times 7} & O_{10\times 7} & A_{2} & O_{10\times 7} & O_{10\times 7} & O_{10\times 7} & O_{10\times 7}\\ O_{10\times 7} & O_{10\times 7} & O_{10\times 7} & A_{2} & O_{10\times 7} & O_{10\times 7} & O_{10\times 7} \end{array}\right]\times X_{2D} \end{array}$$

(29) $$\begin{array}{l} Y_{1}=\left[\begin{array}{ccc} O_{4\times 10} & O_{4\times 10} & O_{4\times 10}\\ A_{0} & A_{0} & A_{0}\\ O_{4\times 10} & A_{0} & O_{4\times 10}\\ O_{4\times 10} & O_{4\times 10} & A_{0} \end{array}\right]\times diag\left(\left[\begin{array}{cccc} O_{10\times 4} & O_{10\times 4} & A_{1} & O_{10\times 4}\\ O_{10\times 4} & A_{1} & A_{1} & O_{10\times 4}\\ P & O_{10\times 4} & A_{1} & O_{10\times 4} \end{array}\right]H^{T}\right)\times \\ \;\times \left[\begin{array}{ccccccc} O_{10\times 7} & -A_{2} & A_{2} & -A_{2} & -A_{2} & O_{10\times 7} & O_{10\times 7}\\ O_{10\times 7} & O_{10\times 7} & O_{10\times 7} & A_{2} & O_{10\times 7} & O_{10\times 7} & O_{10\times 7}\\ O_{10\times 7} & O_{10\times 7} & O_{10\times 7} & O_{10\times 7} & A_{2} & O_{10\times 7} & O_{10\times 7} \end{array}\right]\times X_{2D} \end{array}$$

(30) $$\begin{array}{l} Y_{2}=\left[\begin{array}{ccc} O_{4\times 10} & O_{4\times 10} & O_{4\times 10}\\ O_{4\times 10} & O_{4\times 10} & O_{4\times 10}\\ A_{0} & A_{0} & O_{4\times 10}\\ O_{4\times 10} & A_{0} & A_{0} \end{array}\right]\times diag\left(\left[\begin{array}{cccc} O_{10\times 4} & A_{1} & O_{10\times 4} & O_{10\times 4}\\ A_{1} & A_{1} & O_{10\times 4} & O_{10\times 4}\\ A_{1} & O_{10\times 4} & O_{10\times 4} & O_{10\times 4} \end{array}\right]H^{T}\right)\times \\ \;\times \left[\begin{array}{ccccccc} O_{10\times 7} & O_{10\times 7} & -A_{2} & -A_{2} & A_{2} & -A_{2} & O_{10\times 7}\\ O_{10\times 7} & O_{10\times 7} & O_{10\times 7} & O_{10\times 7} & O_{10\times 7} & A_{2} & O_{10\times 7}\\ O_{10\times 7} & O_{10\times 7} & O_{10\times 7} & -A_{2} & -A_{2} & -A_{2} & A_{2} \end{array}\right]\times X_{2D}^{T} \end{array}$$

in which the block matrices have the form given below:

(31) $$A_{0}=\left[\begin{array}{cccccccccc} 1 & 1 & 1 & 1 & 0 & 0 & 0 & 0 & 0 & 0\\ 0 & 1 & 0 & 0 & 1 & 1 & 1 & 0 & 0 & 0\\ 0 & 0 & 1 & 0 & 0 & 1 & 0 & 1 & 1 & 0\\ 0 & 0 & 0 & 1 & 0 & 0 & 1 & 0 & 1 & 1 \end{array}\right]\;;\;A_{1}=\left[\begin{array}{cccc} 0 & 0 & 0 & 1\\ 0 & 0 & 1 & 1\\ 0 & 1 & 0 & 1\\ 1 & 0 & 0 & 1\\ 0 & 0 & 1 & 0\\ 0 & 1 & 1 & 0\\ 1 & 0 & 1 & 0\\ 0 & 1 & 0 & 0\\ 1 & 1 & 0 & 0\\ 1 & 0 & 0 & 0 \end{array}\right];A_{2}=\left[\begin{array}{ccccccc} 1 & -1 & -1 & -1 & \;\;\;0 & \;\;\;0 & 0\\ 0 & \;\;\;1 & \;\;\;0 & \;\;\;0 & \;\;\;0 & \;\;\;0 & 0\\ 0 & \;\;\;0 & \;\;\;1 & \;\;\;0 & \;\;\;0 & \;\;\;0 & 0\\ 0 & \;\;\;0 & \;\;\;0 & \;\;\;1 & \;\;\;0 & \;\;\;0 & 0\\ 0 & -1 & \;\;\;1 & -1 & -1 & \;\;\;0 & 0\\ 0 & \;\;\;0 & \;\;\;0 & \;\;\;1 & \;\;\;0 & \;\;\;0 & 0\\ 0 & \;\;\;0 & \;\;\;0 & \;\;\;0 & \;\;\;1 & \;\;\;0 & 0\\ 0 & \;\;\;0 & -1 & -1 & \;\;\;1 & -1 & 0\\ 0 & \;\;\;0 & \;\;\;0 & \;\;\;0 & \;\;\;0 & \;\;\;1 & 0\\ 0 & \;\;\;0 & \;\;\;0 & -1 & -1 & -1 & 1 \end{array}\right]$$

and $O_{4\times 10}$, $O_{10\times 4}$, $O_{10\times 7}$ are zero matrices of size 4×10, 10×4, and 10×7, respectively. Adding the partial results $Y_{0}$, $Y_{1}$ and $Y_{2}$ given by (28), (29) and (30), the following output vector Y containing 16 samples of the filtered image is obtained:

(32) $$\begin{array}{l} Y=Y_{0}+Y_{1}+Y_{2}\\ ={\left[\begin{array}{cccccccccccccccc} Y_{00} & Y_{01} & Y_{02} & Y_{03} & Y_{10} & Y_{11} & Y_{12} & Y_{13} & Y_{20} & Y_{21} & Y_{22} & Y_{23} & Y_{30} & Y_{31} & Y_{32} & Y_{33} \end{array}\right]}^{T} \end{array}$$

The vector H occurring in Equations (28)–(30) is given below:

(33) $$H={\left[\begin{array}{cccccccccccccccc} h_{00} & h_{01} & h_{02} & h_{03} & h_{10} & h_{11} & h_{12} & h_{13} & h_{20} & h_{21} & h_{22} & h_{23} & h_{30} & h_{31} & h_{32} & h_{33} \end{array}\right]}^{T}$$

while the input vector $X_{2D}$ is displayed as follows:

(34) $$X_{2D}={\left[\begin{array}{cccccccccccccccc} x_{00} & x_{01} & \ldots & x_{06} & x_{10} & x_{11} & \ldots & x_{16} & \ldots & x_{60} & x_{61} & x_{62} & x_{63} & x_{64} & x_{65} & x_{66} \end{array}\right]}^{T}$$

The main reason for proposing this 2D FIR filtering algorithm was the reduced number of arithmetic operations involved. It is well known that in a direct 2D convolution there is a high degree of redundancy in operations. In a direct 2D convolution there are overlapping blocks of input data; by eliminating these redundant calculations, a significant reduction in the arithmetic complexity will be obtained. The filtering algorithm presented above was produced using a block filtering technique.

At this point, the 2D filtering algorithm discussed above will be extended from an elementary kernel of size 4×4 to the case of a 31 × 31 kernel. In order to achieve this and to obtain a parallel implementation, a block processing technique will be used, relying on a polyphase structure. To derive this 2D polyphase structure, a decimation of the kernel matrix with factor 4 will be performed. Before decimation, the kernel was enlarged to have a dimension multiple of 4, in our case 32 × 32, by bordering it with a row and a column of zeros. Decimation by a factor of 5 was also applied to the input image and thus a 25 × 25 input image was produced.

Using a block polyphase decomposition and the previous fast algorithm, the following efficient algorithm was obtained for the computation of the designed 2D FIR filter. The vectors $H_{00}^{T}$, $H_{01}^{T}$, $H_{02}^{T}$, $H_{03}^{T}$$H_{10}^{T}$, $H_{11}^{T}$, $H_{12}^{T}$, $H_{13}^{T}$$H_{20}^{T}$$H_{21}^{T}$, $H_{22}^{T}$, $H_{23}^{T}$, $H_{30}^{T}$$H_{31}^{T}$, $H_{32}^{T}$, $H_{33}^{T}$ for a kernel matrix of size 12 × 12 and an input matrix of size 21 × 21 have the general form $H_{ij}$ given below (where i=0,1,2,3 and j=0,1,2,3):

(35) $$H_{ij}=\left[\begin{array}{ccccccccc} h_{0+i,0+j} & h_{0+i,4+j} & h_{0+i,8+j} & h_{4+i,0+j} & h_{4+i,4+j} & h_{4+i,8+j} & h_{8+i,0+j} & h_{8+i,4+j} & h_{8+i,8+j} \end{array}\right]$$

For example, the vectors $H_{00}$, $H_{12}$, $H_{33}$ generated by the Formula (35) will be:


(36) $$\begin{array}{l} H_{00}=\left[\begin{array}{ccccccccc} h_{0,0} & h_{0,4} & h_{0,8} & h_{4,0} & h_{4,4} & h_{4,8} & h_{8,0} & h_{8,4} & h_{8,8} \end{array}\right](i=0,\;\;j=0)\\ H_{12}=\left[\begin{array}{ccccccccc} h_{1,2} & h_{1,6} & h_{1,10} & h_{5,2} & h_{5,5} & h_{5,8} & h_{9,2} & h_{9,5} & h_{9,8} \end{array}\right](i=1,\;\;j=2)\\ H_{33}=\left[\begin{array}{ccccccccc} h_{3,3} & h_{3,7} & h_{3,11} & h_{7,3} & h_{7,7} & h_{7,11} & h_{11,3} & h_{11,7} & h_{11,11} \end{array}\right](i=3,\;\;j=3) \end{array}$$

In order to explain the proposed method in an easier way, our demonstration was restricted to a less complex particular situation where the kernel matrix is of size 12 × 12 and the input image is 21 × 21, but the results can be readily extended for the kernel of the circular FIR filter designed above of size 31 × 31, previously extended to size 32 × 32 (by padding with zeros), to be able to achieve the decimation by a factor of 4.

The simpler algorithm described above for a 2D filter with a 3 × 3 kernel and 5 × 5 input matrix, can be extended by performing a decimation by factor 4 for the kernel matrix and a decimation by factor 5 for the input matrix. Thus, performing a decimation with factor 4, instead of the kernel of size 12 × 12, 16 matrices of size 3 × 3 are derived. For instance, in the case of $H_{01}^{T}$, applying decimation by 4, the following block matrix of size 3 × 3 will produce:

(37) $$H_{01}^{\prime }=\left[\begin{array}{ccc} h_{01} & h_{05} & h_{09}\\ h_{41} & h_{45} & h_{49}\\ h_{81} & h_{85} & h_{89} \end{array}\right]$$

Next, by concatenating the rows of matrix $H_{01}^{\prime }$, the matrix $H_{01}^{T}$ is derived from Equation (35). The vector $X_{2D}$ is also substituted with vector $X_{2D}$ given by (34).

The vectors $X_{00}$, $X_{01}$, $X_{02}$, $X_{03}$, …, $X_{66}$, composing the matrix $X_{2D}$ and related to the input image, are defined through the following general formula:

(38) $$X_{ij}=\left[\begin{array}{ccccccccc} x_{14+i,14+j} & x_{14+i,7+j} & x_{14+i,0+j} & x_{7+i,14+j} & x_{7+i,7+j} & x_{7+i,0+j} & x_{0+i,14+j} & x_{0+i,7+j} & x_{0+i,0+j} \end{array}\right]$$

For example, the vectors $X_{03}$, $X_{31}$, $X_{66}$ generated by the Formula (38) will be:


(39) $$\begin{array}{l} X_{03}=\left[\begin{array}{ccccccccc} x_{14,17} & x_{14,10} & x_{14,3} & x_{7,17} & x_{7,10} & x_{7,3} & x_{0,17} & x_{0,10} & x_{0,3} \end{array}\right](i=0,\;\;j=3)\\ X_{31}=\left[\begin{array}{ccccccccc} x_{17,15} & x_{17,8} & x_{17,1} & x_{10,15} & x_{10,8} & x_{10,1} & x_{3,15} & x_{3,8} & x_{3,1} \end{array}\right](i=3,\;\;j=1)\\ X_{66}=\left[\begin{array}{ccccccccc} x_{20,20} & x_{20,13} & x_{20,6} & x_{13,20} & x_{13,13} & x_{13,6} & x_{6,20} & x_{6,13} & x_{6,6} \end{array}\right](i=6,\;\;j=6) \end{array}$$

The vectors $X_{00}$,…, $X_{66}$ were produced as described below. To explain the idea, our demonstration is restricted to the situation in which the input image is a matrix of size 21 × 21 and a decimation by factor 5 is performed. In doing so, instead of the input matrix of dimension 21 × 21, a number of 77 matrices of size 3 × 3 are obtained. For instance, in the case of $X_{01}^{}$, applying decimation by the factor 7, the following 3 × 3 block matrix is derived:

(40) $$X_{01}^{\prime }=\left[\begin{array}{ccc} x_{0,1} & x_{0,8} & x_{0,15}\\ x_{7,1} & x_{7,8} & x_{7,15}\\ x_{14,1} & x_{14,8} & x_{14,15} \end{array}\right]$$

At this point, the rows of matrix $X_{01}^{\prime }$ are concatenated, then the resulting vector is reversed and thus the vector $X_{1,0}$ is derived from the general Equation (38):

(41) $$X_{1,0}=\left[\begin{array}{ccccccccc} x_{15,14} & x_{15,7} & x_{15,0} & x_{8,14} & x_{8,7} & x_{8,0} & x_{1,14} & x_{1,7} & x_{1,0} \end{array}\right]$$

Even if our discussion was restricted to the particular case where the input matrix is 21 × 21 to be easier for the reader to follow our discussion, it is easy to extend it for a more general case. Thus, a 2D FIR filtering operation with a 4 × 4 kernel and a 7 × 7 input matrix was decomposed into 100 1D inner products (FIR filtering operations) using the following equations:


(42) $$\begin{array}{l} Y_{0}=\left[\begin{array}{cccc} B_{0} & B_{0} & B_{0} & B_{0}\\ O_{4\times 90} & B_{0} & O_{4\times 90} & O_{4\times 90}\\ O_{4\times 90} & O_{4\times 90} & B_{0} & O_{4\times 90}\\ O_{4\times 90} & O_{4\times 90} & O_{4\times 90} & B_{0} \end{array}\right]\times diag\left(\left[\begin{array}{cccc} O_{90\times 36} & O_{90\times 36} & O_{90\times 36} & B_{1}\\ O_{90\times 36} & O_{90\times 36} & B_{1} & A_{1}\\ O_{90\times 36} & B_{1} & O_{90\times 36} & A_{1}\\ B_{1} & O_{90\times 36} & O_{90\times 36} & A_{1} \end{array}\right]H_{2}^{T}\right)\times \\ \;\times \left[\begin{array}{ccccccc} B_{2} & -B_{2} & -B_{2} & -B_{2} & O_{90\times 63} & O_{90\times 63} & O_{90\times 63}\\ O_{90\times 63} & B_{2} & O_{90\times 63} & O_{90\times 63} & O_{90\times 63} & O_{90\times 63} & O_{90\times 63}\\ O_{90\times 63} & O_{90\times 63} & B_{2} & O_{90\times 63} & O_{90\times 63} & O_{90\times 63} & O_{90\times 63}\\ O_{90\times 63} & O_{90\times 63} & O_{90\times 63} & B_{2} & O_{90\times 63} & O_{90\times 63} & O_{90\times 63} \end{array}\right]\times X_{2}^{T} \end{array}$$

(43) $$\begin{array}{l} Y_{1}=\left[\begin{array}{ccc} O_{4\times 90} & O_{4\times 90} & O_{4\times 90}\\ B_{0} & B_{0} & B_{0}\\ O_{4\times 90} & B_{0} & O_{4\times 90}\\ O_{4\times 90} & O_{4\times 90} & B_{0} \end{array}\right]\times diag\left(\left[\begin{array}{cccc} O_{90\times 36} & O_{90\times 36} & B_{1} & O_{90\times 36}\\ O_{90\times 36} & B_{1} & B_{1} & O_{90\times 36}\\ B_{1} & O_{90\times 36} & B_{1} & O_{90\times 36} \end{array}\right]H_{2}^{T}\right)\times \\ \;\times \left[\begin{array}{ccccccc} O_{90\times 63} & -B_{2} & B_{2} & -B_{2} & -B_{2} & O_{90\times 63} & O_{90\times 63}\\ O_{90\times 63} & O_{90\times 63} & O_{90\times 63} & B_{2} & O_{90\times 63} & O_{90\times 63} & O_{90\times 63}\\ O_{90\times 63} & O_{90\times 63} & O_{90\times 63} & O_{90\times 63} & B_{2} & O_{90\times 63} & O_{90\times 63} \end{array}\right]\times X_{2}^{T} \end{array}$$

(44) $$\begin{array}{l} Y_{2}=\left[\begin{array}{ccc} O_{4\times 90} & O_{4\times 90} & O_{4\times 90}\\ O_{4\times 90} & O_{4\times 90} & O_{4\times 90}\\ B_{0} & B_{0} & O_{4\times 90}\\ O_{4\times 90} & B_{0} & B_{0} \end{array}\right]\times diag\left(\left[\begin{array}{cccc} O_{90\times 36} & B_{1} & O_{90\times 36} & O_{90\times 36}\\ B_{1} & B_{1} & O_{90\times 36} & O_{90\times 36}\\ B_{1} & O_{90\times 36} & O_{90\times 36} & O_{90\times 36} \end{array}\right]H_{2}^{T}\right)\times \\ \;\times \left[\begin{array}{ccccccc} O_{90\times 63} & O_{90\times 63} & -B_{2} & -B_{2} & B_{2} & -B_{2} & O_{90\times 63}\\ O_{90\times 63} & O_{90\times 63} & O_{90\times 63} & O_{90\times 63} & O_{90\times 63} & B_{2} & O_{90\times 63}\\ O_{90\times 63} & O_{90\times 63} & O_{90\times 63} & -B_{2} & -B_{2} & -B_{2} & B_{2} \end{array}\right]\times X_{2}^{T} \end{array}$$

Finally, the following output vector is produced:

(45) $$\begin{array}{l} Y=Y_{0}+Y_{1}+Y_{2}\\ ={\left[\begin{array}{cccccccccccccccc} Y_{00} & Y_{01} & Y_{02} & Y_{03} & Y_{10} & Y_{11} & Y_{12} & Y_{13} & Y_{20} & Y_{21} & Y_{22} & Y_{23} & Y_{30} & Y_{31} & Y_{32} & Y_{33} \end{array}\right]}^{T} \end{array}$$

In Equations (42)–(44), the block matrices are, respectively: $B_{0}=A_{0}⊗U_{9}$, where the vector $U_{9}$ is $U_{9}=\left[\begin{array}{ccccccccc} 1 & 1 & 1 & 1 & 1 & 1 & 1 & 1 & 1 \end{array}\right]$ and $B_{1}=A_{1}⊗I_{9}$, where $I_{9}$ is the 9×9 identity matrix (with ones on the main diagonal and zeros elsewhere); we also have $B_{2}=A_{2}⊗I_{9}$. The matrices $O_{4\times 90}$, $O_{90\times 36}$ and $O_{90\times 63}$ are zero matrices of size 4 × 90, 90 × 36 and 90 × 63, respectively.

In order to obtain the above equations, we considered a polyphase decomposition of a 1D filter that can compute four samples in parallel using a decimation factor of 4 as:

(46) $$\left[\begin{array}{c} y_{4n}\\ y_{4n+1}\\ y_{4n+2}\\ y_{4n+3} \end{array}\right]=\left[\begin{array}{ccccccc} H_{3} & H_{2} & H_{1} & H_{0} & 0 & 0 & 0\\ 0 & H_{3} & H_{2} & H_{1} & H_{0} & 0 & 0\\ 0 & 0 & H_{3} & H_{2} & H_{1} & H_{0} & 0\\ 0 & 0 & 0 & H_{3} & H_{2} & H_{1} & H_{0} \end{array}\right]\times \left[\begin{array}{c} X_{4n-3}\\ X_{4n-2}\\ X_{4n-1}\\ X_{4n}\\ X_{4n+1}\\ X_{4n+2}\\ X_{4n+3} \end{array}\right]$$

By extending the Equation (46) to 2D and using sub-expression sharing, we obtained Equations (42)–(44). Although at first sight, the matrix equations describing the proposed polyphase implementation may seem very complex, mainly due to their block structure, they actually lead to a very efficient and economic filtering structure, with a high degree of parallelism and therefore with a low computational complexity in terms of number of arithmetic operations. All these equations were verified in Matlab (version R2017a).

## 5. Discussion

The proposed design method for 2D circular filters is entirely analytical, without using any global numerical optimization techniques. Analytical design methods lead to closed-form and parametric filters, with adjustable, tunable frequency responses. To the best of the authors’ knowledge, the analytical design of FIR circular filter banks has not been systematically approached previously by other researchers. As a reference to existing works, analytical techniques for designing 2D filters of IIR type with circular frequency response, including CFBs, have been previously proposed by the first author [33,34,35].

The Gaussian filter was chosen as a prototype for the CFB due to its advantages. It is a smooth function that can be easily approximated by a trigonometric polynomial and can be scaled on the frequency axis to adjust its selectivity. For very selective filters, the Gaussian shape is probably the best choice. Its frequency response is zero-phase; since frequency components will not be phase-shifted, image distortions will not occur. The resulting filters have accurate shapes, with negligible distortions. Moreover, they can be approximated efficiently, leading to low-order filters.

The circular filter bank (CFB) designed in our paper can be compared with other types of filter banks, from a qualitative point of view. The comparative discussion will mainly refer to works [25,26,27], as well-known multiscale pyramidal decomposition methods. Our proposed filter banks, like the steerable pyramid [25], have rotation invariance, while the Laplacian pyramid [26] and wavelet pyramid [27] are not rotationally invariant. Another important aspect regards frequency plane partitioning. While the steerable, wavelet and Laplacian pyramids all split the image spectrum into fixed sub-band regions, the proposed circular FB is flexible, in the sense that the bandwidths of the sub-band regions can be chosen wider or narrower, with adjustable selectivity, depending on application. This is due to the scalability of Gaussian-shaped filters along the frequency axis, which allows us to obtain filters with imposed selectivity starting from the same prototype. In Section 3, a uniform CFB with 11 components was generated, partitioning the image spectrum into concentric ring-shaped sub-bands. Moreover, in some applications, the non-uniform (dyadic) filter bank is also useful from the multi-resolution point of view. Since the energy of an image spectrum is mainly contained in the low-frequency region and decreases towards higher frequencies, the dyadic-type CFB allows for a more uniform energy distribution on frequency bands. Also, the proposed polyphase implementation structure for the filter bank has a lower arithmetic complexity than other implementations found in the literature.

A rigorous comparison in terms of performance with other circular filters found in literature is quite difficult to make. Design approaches like circular filters in [11,12,13] are very different from the one proposed here and lead to filters with other characteristics and purposes, so they are quite difficult to compare exactly with our proposed method.

To summarize, the novelty of the proposed CFB consists of an analytic design method (yielding parametric, closed-form expressions of frequency response), frequency scalability, flexible partitioning of spectrum sub-bands, low order due to efficient approximation and low arithmetic complexity due to polyphase implementation.

The proposed novel implementation technique significantly reduces the number of arithmetic operations required. A short comparison can be made between the direct convolution operation and the proposed filtering method in terms of computational complexity. The 2D filtering of an image of size M×N pixels, with an FIR filter with kernel size m×n implies a 2D convolution between a m×n matrix and a M×N matrix. This means that the filter kernel slides on the horizontal and vertical axes along the image, so for each pixel m×n multiplications are required; therefore the whole 2D filtering would have approximately a complexity of O(MNmn). It is easy to calculate that the total number of additions are $\left(N+n^{2})(M+m^{2}\right)$.

In the simpler case used to exemplify our implementation, the filter kernel has size 12 × 12, while the image is 21 × 21; thus for usual convolution, there will be 63,504 multiplications with 27,225 additions. In our approach, only 100 inner products are used, with 3 × 3 multiplications and 3 × 3 additions for each, that is 100 × 33 = 900 multiplications and 900 additions, plus 12 × 90 additions in the pre-processing stage and 7 × 10 additions in the post-processing stage. As an additional example, for a larger value of the filter kernel where the filter kernel has the size 20 × 20 while the image is 35 × 35, 100 inner products are used, with 5 × 5 multiplications and 5 × 5 additions for each inner product, that is 100 × 5 × 5 = 2500 multiplications and 2500 additions, plus 20 × 90 additions in pre-processing stage and 70 additions in the post-processing stage.

## 6. Conclusions

The proposed design method for 2D circular filter banks is entirely analytical, without using any global numerical optimization. The advantages of the proposed method compared to other works are: it yields a factored 2D frequency response; the designed CFB is parametric, with adjustable characteristics; the CFB components are solved easily for any choice of number of filters and selectivity; the proposed implementation using polyphase and block filtering leads to a low complexity filter structure. This approach solves the problem of designing an adjustable and efficient circular FB with imposed specifications. The obtained results prove that the proposed rotationally invariant filter banks can be used in decomposing a given image into its subband components.

Taking into account the simulation results on test images and the fact that the original image can be reconstructed very accurately at least from a visual, subjective point of view from its component images, the authors intend in future work to study and investigate whether such CFBs (either uniform or non-uniform) could be used in sub-band coding schemes. While practically and intuitively this would seem possible, the required mathematical conditions for perfect reconstruction will have to be investigated rigorously. Regarding the implementation part, the authors will also study how to choose the decimation factors for the input image and the filter kernel, in order to obtain a very efficient, optimal design, and to minimize the number of arithmetic operations.

## Figures and Tables

**Table 1:** Relative sub-band energies (in %) for the 11 images resulting at the output of the uniform CFB.

ER1	55.81594	ER7	2.51503
ER2	14.03036	ER8	1.63033
ER3	8.12698	ER9	1.68105
ER4	6.36161	ER10	1.17531
ER5	4.81307	ER11	1.84367
ER6	3.64377		

**Table 2:** Relative sub-band energies (in %) for the five images resulting at the output of the dyadic CFB.

Image	ER1	ER2	ER3	ER4	ER5
“Trees”	57.925	13.965	18.430	6.268	3.411
“Fields”	68.003	11.767	11.618	5.433	3.179

## Data Availability

Data are contained within the article.
